# Hepatitis C time trends in reported cases and estimates of the hidden population born before 1965, Denmark and Sweden, 1990 to 2020

**DOI:** 10.2807/1560-7917.ES.2022.27.50.2200243

**Published:** 2022-12-15

**Authors:** Peer Brehm Christensen, Birgit Debrabant, Susan Cowan, Kristian Debrabant, Anne Øvrehus, Ann-Sofi Duberg

**Affiliations:** 1 Department of Infectious Diseases Q, Odense University Hospital, Denmark; 2 Clinical Institute, University of Southern Denmark, Odense, Denmark; 3 Department of Mathematics and Computer Science, University of Southern Denmark, Odense, Denmark; 4 Department of Epidemiology, Biostatistics and Biodemography, University of Southern Denmark, Odense, Denmark; 5 Infectious Disease Epidemiology and Prevention, Statens Serum Institut, Copenhagen, Denmark; 6 Department of Infectious Diseases, Faculty of Medicine and Health, School of Medical Sciences, Örebro University, Sweden

**Keywords:** Hepatitis C, national surveillance, epidemiology, mathematical model, population estimate

## Abstract

**Background:**

According to the World Health Organization, hepatitis C virus (HCV) infection should be under control by 2030.

**Aim:**

Our aim was to describe the size and temporal changes in reported cases of chronic HCV infection in Denmark and Sweden and to estimate the size of the hidden (undiagnosed) population born before 1965.

**Methods:**

We extracted all HCV infections reported to national surveillance systems in Denmark and Sweden from 1990 to 2020. Prediction of the size of the hidden HCV-infected population was restricted to the cohort born before 1965 and cases reported up to 2017. We applied a model based on removal sampling from binomial distributions, estimated the yearly probability of diagnosis, and deducted the original HCV-infected population size.

**Results:**

Denmark (clinician-based) reported 10 times fewer hepatitis C cases annually than Sweden (laboratory and clinician-based), peaking in 2007 (n = 425) and 1992 (n = 4,537), respectively. In Denmark, the birth year distribution was monophasic with little change over time. In recent years, Sweden has had a bimodal birth year distribution, suggesting ongoing infection in the young population. In 2017, the total HCV-infected population born before 1965 was estimated at 10,737 living persons (95% confidence interval (CI): 9,744–11,806), including 5,054 undiagnosed, in Denmark and 16,124 (95% CI: 13,639–18,978), including 10,580 undiagnosed, in Sweden.

**Conclusions:**

The reporting of HCV cases in Denmark and Sweden was different. For Denmark, the estimated hidden population was larger than the current national estimate, whereas in Sweden the estimate was in line with the latest published numbers.

**Key public health message**

**What did you want to address in this study?**

How do reported cases of HCV infection differ over time between Denmark and Sweden and is it possible to develop a model for the undiagnosed population, based on the number of reported cases?

**What have we learnt from this study?**

The majority of reported hepatitis C in Denmark are still ‘baby boomers’, i.e. born between 1945 and 1965, in contrast to Sweden where new infections among the young now dominate. Our mathematical model suggests that about half of hepatitis C patients born before 1965 are undiagnosed.

**What are the implications of your findings for public health?**

Most European countries have national surveillance for hepatitis C. These registers can be used to estimate the number of undiagnosed cases based on our model and this may help countries to eliminate hepatitis C.

## Introduction

Hepatitis C virus (HCV) infection remains a major health challenge worldwide, with 57 million chronic infections, leading to 257,000 deaths from decompensated cirrhosis and hepatocellular carcinoma in 2020 [1]. In the European Region, 11.0 million persons (95% confidence interval (CI): 9.0–12 million) are chronically infected, or 1.2% of the population [1]. With the availability of direct-acting antivirals (DAA) since 2014, the World Health Organization (WHO) set goals in 2016 for controlling HCV by 2030. These included 90% reduction in incidence, diagnosis rate of 90%, treatment rate of 80% and 65% reduction in mortality [2].

A major obstacle is that a large proportion of the infected population may not be diagnosed before they present with advanced liver disease [3]. In the European Region, 70.3% of the HCV-infected population was still undiagnosed in 2020 [1]. To prevent transmission, future burden of liver disease and allow national healthcare systems to model future expenditures, it is important to estimate the hidden (i.e. undiagnosed) population with HCV infection and diagnose and treat them before cirrhosis and its complications develop.

The HCV epidemic in high-income countries has largely been driven by unsafe injections among people who inject drugs (PWID) [4,5]. Many methods have been used to estimate and identify the size of the total population of individuals infected with HCV, with the most radical strategy being the universal testing of persons born between 1945 and 1965 (the ‘baby boomer’ generation) in the United States [6-9].

Denmark (population 5.7 million in 2016) [10] and Sweden (population 10.0 million in 2016) [11] are low-endemic countries for HCV infection, with an estimated prevalence of 0.21% in Denmark in 2016 and 0.43% in Sweden in 2014 [12,13]. The proportion of undiagnosed HCV-infected persons is estimated to be 24% in Denmark and 20% in Sweden [12,13]. This hidden population can be seen as two different groups. The first group are people with a long history of undiagnosed HCV infection, some of whom were infected before HCV was discovered. The second group comprises recently infected young PWID with a higher chance of diagnosis in healthcare (e.g. during treatment for drug use or in harm reduction programmes) [14-17].

The Danish HCV reporting system is a national public register of notifiable diseases. For the diagnosing physician, it has been mandatory to report acute hepatitis C since 1991 and chronic HCV infection since May 2000. The case definition from 1991 was a positive antibody test (anti-HCV), and in 2000 a positive HCV RNA test was added. If a case is positive for anti-HCV and/or HCV RNA, it is classified as acute hepatitis C when the infected person has symptoms of acute hepatitis and as chronic hepatitis C when there are no symptoms (unless there was a negative test < 12 months prior). If the case is positive for anti-HCV but an HCV RNA test is missing and the person has no symptoms, the case is classified as chronic. This means that a proportion of past infections (anti-HCV positive/HCV RNA negative) are included as chronic infections in the register. The register is estimated to cover 35–40% of individuals diagnosed with chronic HCV infection [18].

In Sweden, HCV infection became a notifiable disease in 1990 when diagnostic methods became available. Since 1990, it has been mandatory to report both acute and chronic HCV infections based on positive anti-HCV or HCV RNA tests to the Public Health Agency. Initially, only the diagnosing physician had to report but since 1997, there has been a dual notification system with reports from both the physician and the laboratory. In 2020, the case definition changed and only ongoing infection (i.e. HCV RNA-positive) should be reported [19].

Individuals who have been treated and cured will still be in the registers because neither of the countries remove entries when infected persons have been cured. Before 2014, the number of treated persons was < 200 per year in Denmark, increasing to a maximum of 2,000 in 2019. In Sweden, ca 1,000 per year were treated from 2000 to 2014, increasing with new DAA to a maximum of 6,569 in 2018 [13,20,21].

The objective of the present study was to describe the birth year distribution of individuals reported as infected with HCV over time and use this to estimate the size of the hidden HCV-infected populations in Denmark and Sweden. This study focused on the ‘baby boomer’ generation, in which the incidence of new infections was assumed to be low and could be ignored. Our statistical model for estimating the hidden population relied on removal sampling, assuming identical diagnosis probabilities between periods and cohorts, and taking deaths into account.

## Methods

### Settings

Healthcare in the two countries is provided by the state and covers all citizens. Hepatitis C treatment is free of charge and DAA use has been unrestricted since 2018. In both countries, nearly all hepatitis C patients attending clinical care have been treated and cured [20,22].

In both countries, all residents have a personal identification number that is used in all healthcare contacts and national registers, such as the HCV surveillance register. Danish data were extracted from the Danish register for communicable diseases, and Swedish data were provided from the Swedish register for communicable diseases at the Public Health Agency of Sweden [23]. The case definition for Denmark was cases accepted as chronic HCV infection as described above. The case definition for Sweden was cases reported to be anti-HCV-positive. We did not validate the registers. For reported cases of hepatitis C, we extracted year of birth, year of reporting and age at reporting.

### Statistical analysis

Data on HCV infections consist of the number of infected persons reported in a calendar year stratified by 5-year birth cohorts. We extracted all data from 1990 to 2020, but the mathematical model was based on data from 2005 to 2017. This was because the reported numbers for Denmark were small and fluctuated in the first 10–15 years, and a major increase in the number of treated cases was seen in both countries after 2017, when DAA became available without restrictions.

The sampling scheme underlying our data is commonly referred to as removal sampling, and corresponding estimation procedures are frequently applied, especially in the fields of infectious medicine and ecology [24-26]. In essence, data are modelled as successive binomial observations with changing numbers of underlying independent experiments (binomial parameter n) and an unknown success probability in the different experiments (binomial parameter p). In the context of our study, n describes the size of a hidden population and p is the probability of being diagnosed. The classical setup assumes closure of the underlying population and constant (i.e. period-independent) probabilities. Typically, both n and p are unknown and need to be estimated. Our model extends the classical setup by integrating deaths, resulting in a reduction in the underlying population size, and by combining different populations corresponding to different birth cohorts assumed to have the same reporting probabilities but that otherwise correspond to independent removal samples. Details of the statistical model are available in the Supplement.

To estimate the hidden population, we only considered persons born before 1965 and assumed that no new infections occurred in this population after 2005. For each 5-year birth cohort, we estimated the number of undiagnosed HCV-infected persons. Stochastic variation was reduced by combining each consecutive 2-year period of diagnosis into batches, the diagnosis period. To correct for the under-reporting in Denmark, we divided the reported numbers for Denmark by 0.375, assuming a constant reporting coverage during the study period [18]. Death rates were assumed to be age-dependent and based on yearly death rates (cohort data) from the Human Mortality Database [27]. To take HCV-related mortality into account, we used the highest of either the death rate from the mortality database or 0.02 (the observed yearly death rate in the Danish HCV-infected population) [28]. In Sweden, a standardised mortality ratio of 5.8 among HCV-infected persons (compared with the general population) has been reported, and an absolute mortality of 0.027 was used in the model for Sweden [29,30].

Altogether, our model covered nine 5-year birth cohorts (1920–1924, 1925–1930, …, 1960–1964) and the reporting periods of 2005–2006, 2007–2008, etc, up to 2015–2016 in both countries. The initial unknown HCV-infected population (consisting of unknown numbers N_1920–24_,…, N_1960–64_ of individuals from the nine different 5-year birth cohorts) is observed over time. In each diagnosis period, an individual can either be diagnosed or not. We assume that the probability (p) of diagnosis was constant, i.e. independent of the diagnosis period and birth year. Therefore, for each birth cohort and in each diagnosis period, the unknown HCV-infected population is reduced by the number of diagnoses and then further reduced according to the corresponding mortality rate.

Our estimation procedure for the initial unknown size of HCV-infected population (N_1920–24_,…, N_1960–64_) and the detection probability (p) was based on the moment-based regression method [31]. Mortality rates were incorporated by upscaling the number of diagnoses in a period by the product of 1 over the survival probability from previous periods (i.e. as if deaths had not occurred). To take the different birth cohorts into account, we extended the model by a random effect for birth cohorts. Empirical Bayes estimates of random effects were then used to derive the final estimates for N (see the Supplement for the model details).

For the calculation of parametric bootstrap CI, 1,000 artificial diagnosis datasets were simulated based on the estimates derived from the original dataset, and results from re-estimation were combined into CI by the percentile method (Supplement). In a sensitivity analysis, we considered the impact of extending or reducing the considered diagnosis periods and variations in the number of diagnosed cases. We included diagnoses from 2004 and alternatively included diagnoses only from 2006 onwards.

To estimate the number of living persons with a positive HCV test for every 5-year birth cohort and every reporting period, we iteratively updated the number of previously known persons with a positive HCV test by the reported cases in the current reporting period and then reduced the result according to the cohort- and diagnosis period-dependent mortality rate.

## Results

The total number of reported cases in 1990 to 2020 was 10-times higher in Sweden (n = 71,722) than in Denmark (n = 7,219). The maximum number of reported cases in Sweden was in 1992 (n = 4,537), with a second peak in 1997 when laboratory reports were added [32]. The Danish peak was 15 years later and 11 times lower (425 cases in 2007). In both countries, the reported number of cases has been declining and was 1,023 in Sweden and 165 in Denmark in 2020. The observed mortality was 32% of the cohort in Denmark by 2016 and 24% of the cohort in Sweden by the end of 2015 [12,30].

### Birth cohort distributions

**Figure 1:**
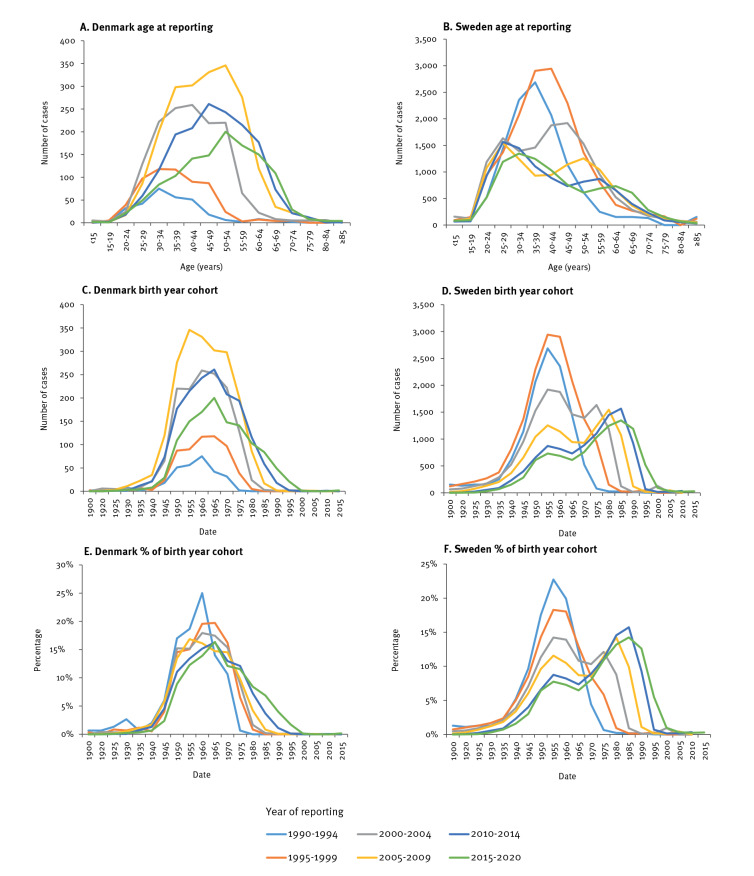
Reported cases of HCV infection by age, birth year and year of reporting, Denmark and Sweden, 1990–2020

The birth years of reported cases with HCV infection show some important differences between Denmark and Sweden (Figure 1).

HCV: hepatitis C virus.

The median birth year among infected persons born before 1980 was 3 years earlier in Sweden than in Denmark (1959 vs 1962). The shape of the birth year distribution was also different between the two countries; in Denmark, there was one stable but wider birth year distribution (1940–1980) with an increasing number of diagnoses by calendar year, peaking in 2005 to 2009. In Sweden, a bimodal curve was evident, with one peak for the ‘baby boomers’ born between 1945 and 1965 mainly diagnosed in the 1990s and then declining over time, and a second peak for the infections reported in the younger population. The number of new diagnoses among ‘baby boomers’ in Sweden has by 2020 become low and has, since the period 2005 to 2009, been lower than the number of diagnoses in persons younger than 30 years (Figure 1). New diagnoses in Sweden are now mainly a result of new infections in the younger population, as demonstrated by the birth year peak advancing consistently with the calendar year of the report. The Swedish observation implies ongoing transmission in the younger population and that a very high proportion of the older population has already been diagnosed. In contrast, Denmark is still reporting the older HCV-infected population, with very few infections reported in the population born after 1980. Correspondingly, during 30 years of reporting to the two registers, the median birth year in Sweden increased by 20 years (1957–1977), whereas it only increased by 6 years (1960–1966) in Denmark.

### Estimates of undiagnosed populations

**Figure 2:**
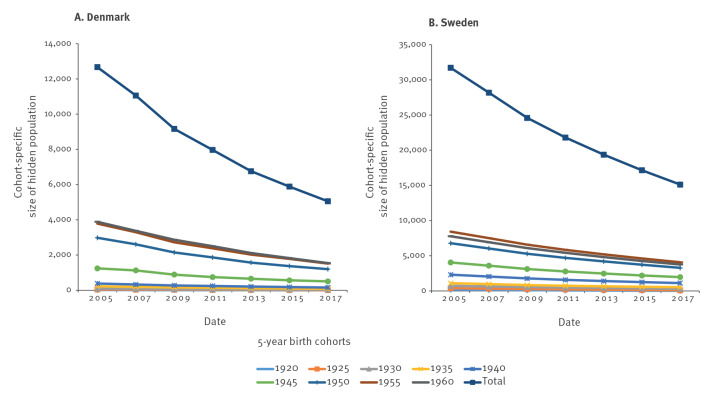
Estimated number of undiagnosed HCV-infected persons born before 1965, by reporting period and by birth cohort, Denmark and Sweden, 1990–2020

For Denmark, the estimated number of undiagnosed cases (birth cohorts 1920–1964, corrected for 37.5% under-reporting) in the beginning of 2017 was 5,054 (95% CI: 4,030–6,156), a decrease from 12,669 (95% CI: 11,166–13,921) in 2005 (Figure 2A).

HCV: hepatitis C virus.

The probability of detecting an undiagnosed infected person within a 2-year period was estimated to be 10.5% (95% CI: 8.9–12.0). Corrected for mortality among the reported cases, this corresponded to a total living HCV-infected population of 12,462 (born 1920–1964) with 41% undiagnosed in Denmark in 2017. Of these 12,462, only 2,778 (22.3%) were identified in the HCV surveillance register. The reduction in the number of HCV-infected cases between 2005 and 2017 was primarily due to individuals dying during the study period. Data on HCV treatment were not available in the register and could not be included in the model. Among the 9% cases reported based on anti-HCV only, 37% will have a past infection, corresponding to 3% of all reported cases [33]. This reduced the 2017 estimate to 12,088. The estimated number of infected persons born before 1965 and cured for HCV in 2006 to 2017 was 1,351; adjusting for this reduced the HCV-infected population to 10,737 (95% CI: 9,744–11,806) with 46% undiagnosed [20].

Using the same model for Sweden for the birth cohort 1920 to 1964, the estimates were 15,114 (95% CI: 11,564–19,191) undiagnosed anti-HCV-positive cases in 2017 and 31,717 (95% CI: 26,425–37,187) in 2005 (Figure 2B). The probability of an unknown infected person being reported in a single 2-year period was estimated to be 6.3% (95% CI: 5.1–7.6). The reduction of 16,603 undiagnosed cases from 2005 to 2017 was due to 9,073 new reported cases and 7,530 deceased. The total number of persons born before 1965 who were living with a past or chronic HCV infection in 2017 was estimated to be 37,946, including 40% undiagnosed. The proportion of chronic infections (HCV RNA-positive) was estimated to be 70%, corresponding to 26,562 [33-36]. Adjusting for infected persons cured between 2006 and 2017 (estimate 10,438), the estimated number of infected persons born before 1965 who were living with chronic HCV infection in Sweden was 16,124 (95% CI: 13,639–18,978), including 40% (10,580) undiagnosed [21].

The sensitivity analysis for the estimation, with diagnosis data from 2004 or 2006 onwards instead of 2005 (i.e. slightly alternative shares of data), resulted in estimated diagnosis probabilities for Denmark of 8.7% and 15.9%, respectively, and 7.9% and 7.4%, respectively, for Sweden.

## Discussion

In this study, we compared the reported cases of HCV infection in Denmark and Sweden and found striking differences between the two countries. Since 1990, Sweden has reported anti-HCV-positive cases, both acute and chronic infections, with the highest number of reports in the 1990s, whereas Denmark mainly reported HCV RNA-positive cases and did not start to report chronic infections until the year 2000. Although anti-HCV-positive cases are still accepted in Denmark if an HCV RNA result is not available, this has only been seen in 9% of reported cases since 2007 (data not shown). If only HCV RNA-positive cases were accepted in Sweden, the prevalence of registered cases would decrease by 23–38% when estimating chronic HCV infections [33-36]. Another important difference is the method of reporting. Since 1997, Sweden has had a dual reporting system with both laboratory and clinical reports, with the number of laboratory reports exceeding the clinical reports by ca 10%. However, from 1990 to 1996, the system was based only on clinical reports, though with high coverage. In contrast, in Denmark, the clinician-based system is estimated to report only 37.5% of cases [18,37]. In addition, Danish cases may be reported years later than the laboratory diagnosis, typically when the infected person is referred for specialist care. This will delay the identification of changes over time. Though the HCV RNA case definition and reporting coverage will not influence the shape of the birth cohort graph of the reported cases, the delay from diagnosis to reporting will result in curve differences if the birth year HCV distribution changes over time, as observed in Sweden.

Taking all reporting differences together, we would expect Denmark to have only one ninth (11%) of the reported numbers in Sweden if the population prevalence was identical. We observed 10.1% (7,219/71,722), supporting that the prevalences in the two countries are in the same range despite the large difference in reported cases [13,33]. We think that Denmark could double the number of reported cases by implementing laboratory reporting of viral hepatitis, whereas implementing HCV RNA reporting in Sweden would allow differentiation between ongoing and past infection at the date of diagnosis. This was introduced in Sweden in 2020 [19].

In Denmark, there was one stable peak around the birth year 1960, suggesting that Denmark is still mainly detecting infections in the ‘baby boomer’ cohort. In Sweden a bimodal curve is evident with one peak around birth year 1955 which has continuously declined and is now very low, revealing a second peak corresponding to persons aged around 30 years. This peak is changing with calendar time and is not fixed to a birth cohort, suggesting ongoing transmission in a certain age group and reflecting new incident cases among young PWID [23]. Interestingly, a shift from a monophasic to a biphasic curve among reported HCV cases in a population over time has previously been reported in the United States, in Massachusetts from 2002 to 2009, coinciding with an outbreak of HCV infection among 15–24-year-olds [38].

The decreasing number of new diagnoses in the older population probably indicates that a large proportion has been diagnosed. A recent Swedish study of pregnant women and their partners (mean age: 30 years) demonstrated an HCV RNA prevalence of 0.4%, and only 17% were not previously diagnosed [17]. Among all estimated HCV infections in Denmark, 54% had been recorded in at least one of four national registers by 2007 and 76% by 2016, suggesting that the proportion of HCV-infected persons identified in the registers has increased in this period [12,33,39,40].

From the current literature and ongoing surveys, we believe that the incidence of HCV infection is decreasing in Denmark primarily because of a switch from injecting to smoking and snorting drugs in the drug-using population [6,40,41]. In Sweden, the incidence seems to be declining more slowly because of a higher incidence among young people. However, the difference may also reflect the much more widespread use of opioid substitution treatment and needle exchange programmes in Denmark and the high proportion of amphetamine use in Sweden, a drug for which no good substitution treatment is available. Finally, the monophasic curve in Denmark could reflect the delay between diagnosis and reporting in the Danish system. However, from the yearly reports of HCV in Denmark in 2018 to 2020, there is still no sign of a bimodal curve and a reporting delay of more than 10 years in Denmark would be necessary to explain the observed difference between the two countries.

Our Danish estimate of 10,737 HCV-infected individuals born before 1965 is twice as high as the latest published estimate in 2016 of 5,287 individuals born before 1965 [12]. The Swedish estimate in 2017 of 16,124 HCV-infected individuals born before 1965 is close to the 2017 estimate in a recent study (total 32,793 HCV infections, of whom we estimate 18,692 (57%) to be born before 1965, based on the age distribution of reported cases in our study) [42].

This study has some limitations. Firstly, reporting systems and case definitions were not the same in the two countries. This is relevant when considering burden of disease, treatment and health economics. In our model, we adjusted for under-reporting in Denmark. This was not necessary in Sweden where surveillance was based on laboratory reporting. Secondly, our estimation procedure did not separate date of diagnosis from date of reporting. By design, the time to reporting was longer in Denmark, where doctor’s delay was a major factor. Another uncertainty was that we could only estimate the population cured for hepatitis C from available treatment data because this is not reported in the current notification system. The large proportion cured with DAA since treatment became universally available in 2018 must be included in future estimates in order to monitor the fulfilment of the WHO HCV elimination strategy by 2030. Therefore, we suggest that the curing of HCV-infected persons should be incorporated in the national surveillance system.

Furthermore, estimating unknown population sizes with unknown detection probabilities has the inherent weakness that large undiagnosed populations with low detection probabilities and small populations with high detection probabilities result in similar reporting frequencies. This is reflected in the relatively wide CI for the hidden population estimates in our model. Moreover, further deviations from model assumptions, such as a non-constant detection probability or presence of new infections, can give rise to bias and fluctuations in estimates for both hidden populations and detection probability. The Swedish data suggest that the younger PWID were driving the HCV epidemic. However, including the population < 40 years of age, with a significant incidence of new infections, undermined the stability of our estimate considerably, and we had to refrain from this to maintain a simple statistical model. 

As negative tests were not reported in either country, we were unable to validate whether testing rates were constant over calendar time and between the two countries. If there was a disproportionately lower testing rate among young drug users in Denmark compared with Sweden, this could explain our results. The sensitivity analysis for the Danish data also indicated possible deviations from model assumptions. Transmission seems to be reported at a higher rate in Sweden than in Denmark, and this may violate the assumption of a negligible number of infections in those older than 40 years. In 2017, 61% of Danish cases, but only 34% of Swedish cases, were older than 40 years. Furthermore, even before the DAA era, Sweden cured a much higher proportion of HCV-infected persons [20,21]. After correcting for cured persons, our estimates were much closer to the previously reported numbers and the Swedish model was more stable than the Danish model.

## Conclusion

We found important differences in the national surveillance systems and the number of reported cases of HCV infection between two otherwise comparable countries. Sweden had a significantly higher proportion of young HCV-infected persons reported to the surveillance register than Denmark. Moreover, we set up an estimation method for the size of the hidden population with HCV infection from the registers of reported cases, as well as for the detection probability. Application of this method to other countries could be useful, as these data are available in many European countries. Better reporting and better knowledge about the population affected by hepatitis C and its size will help us reach the WHO HCV elimination target by 2030.

## Supplementary Data

349000 Supplement
