# Supplementary material for: Hepatitis C time trends in reported cases and estimates of the hidden population born before 1965, Denmark and Sweden, 1990 to 2020
Source: Euro Surveill. 2022 Dec 15;27(50):2200243. doi: 10.2807/1560-7917.ES.2022.27.50.2200243 (PMC9808318; doi:10.2807/1560-7917.ES.2022.27.50.2200243)
Supplement: Supplement [file 22-00243_CHRISTENSEN_Supplement.pdf]

Supplement S1

Hepatitis C in Denmark and Sweden:

Time trends in reported cases and estimates of the hidden population born prior to 1965

"This supplementary material is hosted by Eurosurveillance as supporting information alongside the article, on behalf of the authors, who remain responsible for the accuracy and appropriateness of the content. The same standards for ethics, copyright, attributions and permissions as for the article apply. Supplements are not edited by Eurosurveillance and the journal is not responsible for the maintenance of any links or email addresses provided therein."

Estimation of cohort specific population sizes with emigration and a common diagnosis probability This appendix describes the estimation approach used to estimate the hidden population sizes and gives details on the calculation of confidence intervals:

Notations: In the sequel, we use the following notations:

- N_0^l initial hidden population (unknown, deterministic) from cohort l, $l = 1, \dots, L$, where L is the number of cohorts
- N_j^l hidden population (unknown, random) from cohort l at the end of diagnosis period j for $l = 1, \dots, L$, $j = 1, 2, \dots J$, where J is the number of diagnosis periods
- y_j^l number of diagnoses (observed, random) from cohort l in period j, $j = 1, 2, \dots J$, $l = 1, \dots, L$
- p detection probability (unknown, assumed to be constant)
- α_j^l ($\bar{\alpha}_j^l$) known probability of death (survival) for cohort l in period j, $j = 1, \dots J$, $l = 1, \dots, L$

Note that in this appendix we refer to cohorts instead of cohort batches to enhance understanding. (In relation to the main manuscript, cohorts should be interpreted as cohort batches.)

Modelling: For each period j and each cohort l, we assume that

- diagnoses as well as deaths happen independently for each individual,
- each individual has the same probability p to be diagnosed, and
- diagnoses precede deaths.

This implies the following probabilistic two-step model:

$$\begin{aligned} y_{j+1}^l | N_j^l &\sim Bin(N_j^l, p) \qquad (1) \\ N_{j+1}^l | N_j^l, y_{j+1}^l &\sim Bin\left(N_j^l - y_{j+1}^l, 1 - \alpha_{j+1}^l\right), \quad j = 1, \dots, J, l = 1, \dots, L, \end{aligned}$$

where $\sim$ denotes "is distributed as", and where $Bin(n, p)$ denotes a binomial distribution with parameters n and p.

Estimation: Our procedure aims to simultaneously estimate the initial sizes of the hidden populations and the diagnosis probability, i. e. the unknown parameters N_0^l, $l = 1, \ldots, L$, and p. Death-/survival probabilities α_j^l, $\bar{\alpha}_j^l$, $j = 1, \ldots, J$, $l = 1, \ldots, L$, are assumed to be known and were derived from the ones in Human Mortality Database, ensuring that the death probabilities at least were equal to the yearly observed death rates induced by HCV in Denmark (0.02) respectively Sweden (0.027).

We use a moment-based estimation procedure, which extends the estimation method described in (Zippin, Biometrics, 1956, 163-189) in order to allow for simultaneous integration of several birth cohorts as well as mortality.

Firstly, note that the expected number of diagnoses y_{j+1}^l given all previous diagnoses $y_1^l, \ldots, y_j^l$ is given by

$$E(y_{j+1}^l | y_1^l, y_2^l, \ldots, y_j^l) = N_0^l \prod_{i=1}^{j} \bar{\alpha}_i^l p - \sum_{i=1}^{j} y_i^l \prod_{k=i}^{j} \bar{\alpha}_k^l p,$$

for j=1,2, ..., J, l=1, ..., L.

Considering diagnoses transformed (upscaled) to their hypothetical value if death was impossible, $\tilde{y}_{j+1}^l := \frac{y_{j+1}^l}{\prod_{i=1}^{j} \bar{\alpha}_i^l}$ together with their accumulated values $\tilde{x}_{j+1}^l := \sum_{i=1}^{j} \tilde{y}_i^l$, leads to the following equation:

$$E(\tilde{y}_{j+1}^l | \tilde{y}_1^l, \tilde{y}_2^l, \ldots, \tilde{y}_j^l) = E(\tilde{y}_{j+1}^l | \tilde{x}_{j+1}^l) = N_0^l p - \tilde{x}_{j+1}^l p$$

This is the regression equation of the (scaled) number of diagnoses in a period on the accumulated (scaled) number of diagnoses in previous periods. Given pairs $(\tilde{y}_1^l, \tilde{x}_1^l)$, ..., $(\tilde{y}_J^l, \tilde{x}_J^l)$, one can derive estimates for N_0^l and p from least square estimators for the slope and intercept of the regression line, where especially p is estimated by the absolute value of the regression slope and an estimate for the initial hidden population can be obtained as intercept divided by the estimate for p.

To combine information from all cohorts simultaneously in order to estimate $N_0^1, \ldots, N_0^L$ and a common diagnosis probability p, we used a random effects model

$$\widetilde{y}_{j,l} = A + B\widetilde{x}_{j,l} + u_l + \varepsilon_{j,l}, \qquad j = 1, \ldots, J, l = 1, \ldots, L, \tag{2}$$

where $u_1, \ldots, u_L$ are assumed iid normal random variables (the random intercepts), and $\varepsilon_{j,l}$ are assumed iid normal random residuals. Estimates for p can be derived from estimates for B and estimates for N_0^l can be derived from estimates for A together with predictions for u_l, $l = 1, \ldots, L$.

Estimation algorithm:

- Calculate the scaled number of diagnoses $\tilde{y}_j^l$ as well as the corresponding accumulated diagnoses $\tilde{x}_j^l$ for $j = 1, \dots, J$ and $l = 1, \dots, L$.

- Fit model (2) by maximum likelihood to find estimates $\hat{A}$ and $\hat{B}$ and calculate best linear unbiased predictors (BLUP) $\hat{u}_1, \dots, \hat{u}_L$ for the random effects.

- Estimate the diagnosis probability p by $\hat{p} := -\hat{B}$.

- Estimate the hidden population N_0^l for cohort $l = 1, \dots, L$ by $\hat{N}_0^l := -\frac{\hat{A}+\hat{u}_l}{\hat{B}}$.

Parametric bootstrap confidence intervals: Due to the moment-based estimation approach, confidence intervals were constructed using the following parametric bootstrap approach:

- From the observed data y_j^l, $j = 1, \dots, J$, $l = 1, \dots, L$, obtain estimates $\hat{p}$ and $\hat{N}_0^l$, $l = 1, \dots, L$.

- Repeat BS times:
  - Generate new data from model (1) using parameters $\hat{p}$ and $\hat{N}_0^l$, $l = 1, \dots, L$
  - Calculate estimates ${}^*\hat{p}$ and ${}^*\hat{N}_0^l$, $l = 1, \dots, L$

- calculate bootstrap confidence intervals using the normal approximation
